# Supplementary material for: Chronic bronchitis without airflow obstruction, asthma and rhinitis are differently associated with cardiovascular risk factors and diseases
Source: PLoS One. 2019 Nov 7;14(11):e0224999. doi: 10.1371/journal.pone.0224999 (PMC6837508; doi:10.1371/journal.pone.0224999)
Supplement: Attachment — Submitted filename: response-to-comments-R1.docx [file pone.0224999.s002.docx]

PONE-D-19-23787

Chronic bronchitis without airflow obstruction, asthma and rhinitis are differently associated with cardiovascular risk factors and diseases

**POINT-BY-POINT REPLY TO COMMENTS**

Due date: December 1st 2019

**Journal Requirements**

1) Please ensure that your manuscript meets PLOS ONE's style requirements, including those for file naming. The PLOS ONE style templates can be found at

http://www.journals.plos.org/plosone/s/file?id=wjVg/PLOSOne_formatting_sample_main_body.pdf and http://www.journals.plos.org/plosone/s/file?id=ba62/PLOSOne_formatting_sample_title_authors_affiliations.pdf

We have revised the style of the manuscript to fulfil PLOS ONE requirements, including title page requirements, labelling of Figures, file naming, and reference formatting.

2) Thank you for including your ethics statement; "Ethical approval was obtained in each centre from the appropriate ethics committee, and written consent was obtained from each participant (see Acknowledgements)." Please amend your current ethics statement to include the full name of the ethics committee/institutional review board(s) that approved your specific study. Once you have amended this/these statement(s) in the Methods section of the manuscript, please add the same text to the “Ethics Statement” field of the submission form (via “Edit Submission”).

We have implemented this change in the manuscript (lines 106-112 of the tracked-changes version) and amended the ethics statement in the online submission form.

3) We note that you have indicated that data from this study are available upon request. PLOS only allows data to be available upon request if there are legal or ethical restrictions on sharing data publicly. For information on unacceptable data access restrictions, please see http://journals.plos.org/plosone/s/data-availability#loc-unacceptable-data-access-restrictions.

In your revised cover letter, please address the following prompts:

a) If there are ethical or legal restrictions on sharing a de-identified data set, please explain them in detail (e.g., data contain potentially identifying or sensitive patient information) and who has imposed them (e.g., an ethics committee). Please also provide contact information for a data access committee, ethics committee, or other institutional body to which data requests may be sent.

b) If there are no restrictions, please upload the minimal anonymized data set necessary to replicate your study findings as either Supporting Information files or to a stable, public repository and provide us with the relevant URLs, DOIs, or accession numbers. Please see http://www.bmj.com/content/340/bmj.c181.long for guidelines on how to de-identify and prepare clinical data for publication. For a list of acceptable repositories, please see http://journals.plos.org/plosone/s/data-availability#loc-recommended-repositories.

We will update your Data Availability statement on your behalf to reflect the information you provide.

We have included a “minimal anonymised data set” as an additional online supplementary file (S1 Dataset), and updated our data availability statement accordingly.

4) While revising your submission, please upload your figure files to the Preflight Analysis and Conversion Engine (PACE) digital diagnostic tool, https://pacev2.apexcovantage.com/. PACE helps ensure that figures meet PLOS requirements. To use PACE, you must first register as a user. Registration is free. Then, login and navigate to the UPLOAD tab, where you will find detailed instructions on how to use the tool. If you encounter any issues or have any questions when using PACE, please email us at figures@plos.org. Please note that Supporting Information files do not need this step.

We have checked the figure files using PACE as requested.

**Reviewers' comments**

1) My major concern is a definition of the groups. For CB from the text I understand that their lung function was normal. That should be stated in the definition.

Thanks for your comment. We have revised the definition of chronic bronchitis as suggested (line 132 in the tracked-changes version of the manuscript).

2) Also since they were tested to allergies and metacholine was that an exclusion criteria? If yes that should be stated in the definition and an explanation of what caused CB since they were younger and 44% non-smokers?

As reported in the Methods section, we adopted a hierarchical classification of cases (lines 259-261). As a consequence, chronic bronchitis has cough and phlegm without bronchial obstruction as a hallmark, but in this group also subjects with asthma, rhinitis or atopy may be present. Positive skin prick tests or positive methacholine challenge were not exclusion criteria.

In the group of subjects with chronic bronchitis, the symptoms may be related to causes other than smoking, including e.g. post-nasal drip and airway inflammation of asthma. Moreover, we cannot rule out that the symptoms can be linked to air pollution, which is a major issue in the Po Valley (Verona, Pavia and Turin centres). As highlighted in the manuscript, our findings suggest the critical clinical relevance of cough and phlegm even in the absence of smoking.

3) Asthma group shouldn’t be defined by 1. Criteria, only the second criteria is excluding non asthmatics from the group.

“Definition 1” is “self-reported asthma, plus one among having had an asthma attack in the last 12 months or current use of medications for asthma”. This epidemiological disease definition has been widely used, and validated in studies demonstrating an excellent specificity, 99.7% (de Marco R et al. Eur Respir J. 1998;11: 599-605).

4) Where are asthma and CB patients with rhinitis? Are they in asthma and CB groups (should be stated how much) or excluded?

Due to our hierarchical definitions, subjects with rhinitis could be included either among cases of chronic bronchitis, or among cases of asthma, or among cases of rhinitis alone. This is explained in lines 131-140 of the manuscript.

In the revised manuscript, we have added the following sentence to the results section (lines 192-194): “As a result of our hierarchical definitions, cases of chronic bronchitis could also be affected by asthma (n=115, 45.3%) as well as rhinitis (n=205, 80.7%), and cases of asthma could also be affected by rhinitis (n=354, 84.7%)”.

**We thank the reviewer for the time he/she dedicated in reviewing the manuscript.**
